# Improvement in Multi-Angle Plane Wave Image Quality Using Minimum Variance Beamforming with Adaptive Signal Coherence

**DOI:** 10.3390/s24010262

**Published:** 2024-01-02

**Authors:** Che-Chou Shen, Chun-Lin Huang

**Affiliations:** Department of Electrical Engineering, National Taiwan University of Science and Technology, Taipei 106335, Taiwan

**Keywords:** plane wave imaging, signal coherence, delay-multiply-and-sum, minimum variance, adaptive delay-multiply-and-sum

## Abstract

For ultrasound multi-angle plane wave (PW) imaging, the coherent PW compounding (CPWC) method provides limited image quality because of its conventional delay-and-sum beamforming. The delay-multiply-and-sum (DMAS) method is a coherence-based algorithm that improves image quality by introducing signal coherence among either receiving channels or PW transmit angles into the image output. The degree of signal coherence in DMAS is conventionally a global value for the entire image and thus the image resolution and contrast in the target region improves at the cost of speckle quality in the background region. In this study, the adaptive DMAS (ADMAS) is proposed such that the degree of signal coherence relies on the local characteristics of the image region to maintain the background speckle quality and the corresponding contrast-to-noise ratio (CNR). Subsequently, the ADMAS algorithm is further combined with minimum variance (MV) beamforming to increase the image resolution. The optimal MV estimation is determined to be in the direction of the PW transmit angle (Tx) for multi-angle PW imaging. Our results show that, using the PICMUS dataset, TxMV-ADMAS beamforming significantly improves the image quality compared with CPWC. When the *p* value is globally fixed to 2 as in conventional DMAS, though the main-lobe width and the image contrast in the experiments improve from 0.57 mm and 27.0 dB in CPWC, respectively, to 0.24 mm and 38.0 dB, the corresponding CNR decreases from 12.8 to 11.3 due to the degraded speckle quality. With the proposed ADMAS algorithm, however, the adaptive *p* value in DMAS beamforming helps to restore the CNR value to the same level of CPWC while the improvement in image resolution and contrast remains evident.

## 1. Introduction

Medical ultrasound (US) imaging has been widely used in clinical applications due to its real-time and cost-efficient capabilities. Conventionally, US imaging depends on the focused transmission of the acoustic beam to suppress off-axis clutters in transmission. The downside of focused transmission is that only a small part of the imaged target is illuminated in each beam and thus the entire field of view of the image has to be constructed using multiple focused transmissions. According to the spatial sampling rate, the number of focused beams for one US image typically ranges from one hundred to two hundred. This significantly limits the achievable temporal resolution (i.e., frame rate) of conventional US imaging. In plane wave (PW) imaging, on the contrary, the unfocused beam is used to illuminate the tissue with a wide field of view. With only a single PW transmission, one US image can be produced by a coherent sum of the received channel data after time compensation for the geometric propagation paths from each image pixel [1]. Note that the coherent sum of delayed channel data is generally referred to as delay-and-sum (DAS) beamforming [2]. Since DAS beamforming in theory has limited image resolution and insufficient rejection of off-axis clutter, the corresponding PW imaging inevitably suffers from an evident loss of image quality due to the lack of transmit focusing. Coherent plane wave compounding (CPWC) improves the image quality of PW imaging by collecting low-quality images with different PW transmit angles and then coherently compounds these low-quality images to form the final high-quality image [3,4]. Note that CPWC imaging is still constructed via DAS beamforming of the corresponding two-dimensional (2D) echo matrix with the PW transmit angle and the receiving channel in each dimension. Here, the sum of received channel data corresponds to dynamic receive focusing while the compounding of different PW transmit angles achieves synthetic transmit focusing. To boost the dynamic receive focusing in CPWC, the aperture size can be physically enlarged by combining multiple arrays [5]. To provide synthetic transmit focusing with a minimal number of PW transmit angles, deep learning-based beamforming methods have also been proposed to reconstruct the PW image from a pre-trained convolutional neural network [6,7,8,9,10]. Nonetheless, the image quality of CPWC is still compromised due to the data-independent characteristic of DAS beamforming.

Various data-dependent imaging methods have been proposed to provide adaptive enhancement of US image quality. In minimum variance (MV) beamforming [11,12,13], the covariance matrix of the signals has to be first estimated to obtain the MV weights via matrix inversion. Compared with the conventional DAS method in which the data array is apodized using a fixed weighting function in the coherent summation regardless of the feature of received data, the MV weights are calculated by minimizing the variance in the beamforming response (i.e., maximally narrowing the radiation pattern). Therefore, the MV weights can suppress the presence of off-axis clutters and thus improve image quality. Eigenspace-based MV is also developed by enhancing the covariance matrix for MV estimation using eigendecomposition into signal subspace [14,15,16]. Many MV-based beamforming methods have been specifically developed for multi-angle PW imaging; joint transmit–receive (JTR) beamforming calculates MV weights in both dimensions of the PW transmit angle and receiving channel for the echo matrix with sub-matrix averaging [17]. In double MV, the first MV estimation is applied in the direction of the receiving channel for each low-quality image and then the second MV estimation is applied to these low-quality images in the direction of the PW transmit angle [18]. DCR-MVDR beamforming has also been developed without subarray averaging by generating the snapshots for the covariance matrix estimation from a set of vectors obtained by slightly different compounding of the 2D echo matrix [19]. Since DCR-MVDR utilizes the full size of a 2D echo matrix for decorrelation, it is reported to have better image resolution and contrast than conventional MV methods.

MV beamforming has also been combined with coherence-based adaptive weighting and compressive sensing to further suppress spatially incoherent clutters [20,21,22,23,24]. The signal coherence is generally estimated by using the coherence factor [25,26], which is based on the power ratio of the coherent signal to the incoherent clutters as the pixel-wise weighting of the CPWC image. The short-lag spatial coherence (SLSC) beamforming method [27,28] is also a coherence-based technique that estimates the spatial coherence of backscattered echoes and uses this information to directly form an image. This approach has shown superior lesion detection performance in conventional B-mode imaging. Recently, a novel nonlinear beamforming algorithm called delay-multiply-and-sum (DMAS) has been proposed for ultrasound imaging [29,30]. The DMAS algorithm involves multiplying channel echoes in pairs to introduce spatial coherence into the beamforming process for effective suppression of side-lobe clutter and ghost artifacts in the output. Its high-order version of DMAS beamforming further allows flexible manipulation of the extent of spatial coherence in the image output [31,32]. Specifically, for any rational *p* value larger than unity, the DMAS algorithm can be efficiently implemented by taking the *p*-th root of the echo magnitude and then the *p*-th power after the coherent summation to restore the signal dimensionality. Note that, when a higher *p* value is used in DMAS beamforming, the image output is more modulated by the extent of signal coherence. Consequently, for a diffused speckle background with random signal coherence, the speckle variation would be markedly elevated in DMAS beamforming with a high *p* value and thus the corresponding speckle quality degrades [33].

In our previous study [34], MV-DMAS beamforming combining MV with DMAS was developed for conventional US imaging with focused transmission. The results show that, since DMAS provides good clutter suppression capability while MV reduces off-axis scattering energy, the integrated MV-DMAS beamforming method significantly improves both image resolution and contrast at the same time. Inspired by these findings, we preliminarily developed an alternative version of MV-DMAS beamforming specifically for multi-angle PW imaging at the conference of IUS 2022 [35] and confirmed that the image resolution and contrast can be improved but at the cost of speckle quality in the background region. The degradation of speckle quality results from the intrinsic feature of the conventional DMAS algorithm in which a global *p* value is assigned to every image pixel regardless of its characteristics [36]. In this study, the performance of various MV beamforming methods in multi-angle PW imaging is firstly examined without considering the DMAS algorithm in order to find the optimal MV beamforming strategy in multi-angle PW imaging. Then, an adaptive *p* value in the DMAS algorithm (ADMAS) is further proposed in this study to simultaneously allow for a high *p* value to suppress clutter artifacts and a low *p* value to preserve the speckle background. This article is organized as follows. In Section 2, the theoretical background of the MV beamforming methods used for comparison in this study is introduced. The integration of MV beamforming with the DMAS algorithm is also explained in detail. In Section 3, research methods such as simulation and experimental setups are provided with quantitative image quality metrics. The results are presented in Section 4. In Section 5, a brief conclusion of the entire article is provided and discussed.

## 2. Theory

### 2.1. CPWC Beamforming

Assuming that there are *N* receiving channels for *M* transmissions to different PW angles, the data for the *n*-th depth sample of the image can be time-compensated according to the geometric propagation path and arranged in a 2D echo matrix X(n). The echo matrix X(n) can be expressed as follows:

(1) $$X(n)=\left[\begin{array}{cccc} x_{1,1}(n) & x_{1,2}(n) & \cdots & x_{1,N}(n)\\ x_{2,1}(n) & x_{2,2}(n) & \cdots & x_{2,N}(n)\\ \vdots & \vdots & \ddots & \vdots \\ x_{M,1}(n) & x_{M,2}(n) & \cdots & x_{M,N}(n) \end{array}\right]$$

where $x_{i,j}(n)$ represents the time-compensated data in the *j*-th receiving channel of the *i*-th PW transmit angle. Note that X(n) is often synthesized along one of its dimensions to reduce the amount of data for subsequent beamforming. For instance, when X(n) is compounded along the direction of the receiving channel, the signal at each transmit angle can be written as:

(2) $$Z(n)={\left[z_{1}(n)z_{2}(n)z_{3}(n)\;\ldots z_{M}(n)\right]}^{T}$$

where

$$z_{i}(n)=\frac{1}{N}\sum_{j=1}^{N}x_{i,j}(n),\;\mathrm{for}\;i=1\sim M.$$

Here, Z(n) is referred to as the Tx angle array after the channel sum in this study. Note that the elements of Z(n) are actually low-resolution data obtained using a single PW transmitter. On the contrary, when X(n) is compounded along the direction of the PW transmit angle, the synthetic transmit signal at each receiving channel can be written as:

(3) $$V(n)={\left[v_{1}(n)v_{2}(n)v_{3}(n)\;\ldots v_{N}(n)\right]}^{T}$$

where

$$v_{j}(n)=\frac{1}{M}\sum_{i=1}^{M}x_{i,j}(n),\;\mathrm{for}\;j=1\sim N$$

Here, V(n) is referred to as the Rx channel array after synthetic transmit focusing in this study. With the aforementioned data array in (2) and (3), the CPWC beamforming output can be represented as the dot product of the data array and a uniform weighting vector as shown in the following:

(4) $$y_{CPWC}(n)=w_{T}^{H}(n)Z(n)=w_{R}^{H}(n)V(n)$$

where ${(.)}^{H}$ denotes the conjugate transpose. Note that $w_{T}^{}=[1,1,\ldots {,1]}^{T}/M$ and $w_{R}^{}=[1,1,\ldots {,1]}^{T}/N$ are, respectively, the uniform weighting vectors for the Tx angle array and the Rx channel array.

### 2.2. MV Beamforming for PW Imaging

MV beamforming improves the image resolution by minimizing the variance in the beamformer output with data-dependent weighting. The weighting vector could be optimized through the following constrained optimization problem:

(5) $$\min\;w^{H}(n)R(n)w(n)\;s.t.\;w^{H}(n)a=1$$

where R(n) is the auto-covariance matrix of the data array from either Z(n) or V(n), which is, respectively, referred to as TxMV and RxMV beamforming in this study. The analytical solution to this optimization problem can be calculated by using the Lagrange method as follows:

(6) $$w(n)=\frac{R{\left(n\right)}^{-1}a}{a^{H}R{\left(n\right)}^{-1}a}$$

In ultrasound imaging, since the data vector has been time-compensated for each image pixel to align the data to the main-lobe direction, the steering vector a can be simplified to a constant vector comprised of elements whose value is one. Moreover, in order to ensure the estimation of the auto-covariance matrix is robust and invertible, spatial and temporal averaging is generally applied together with diagonal loading. In RxMV beamforming, the MV estimation is performed on the Rx channel array (i.e., V(n)) with spatial averaging to improve the robustness of the MV estimation. The TxMV beamformer is generally similar to the RxMV counterpart but with two differences. The first difference is that the MV estimation is performed on the Tx angle array (i.e., Z(n)). The second difference is that the temporal averaging of the auto-covariance matrix can be performed in the TxMV estimation to further restore the background speckle quality. The corresponding averaged auto-covariance matrix in the forward direction can then be estimated as:

(7) $$\begin{array}{l} R_{V}^{}(n)=\frac{1}{N-L+1}\sum_{l=1}^{N-L+1}V_{k}(n)\cdot V_{k}{}^{H}(n)\\ R_{Z}(n)=\frac{1}{\left(2T+1\right)\left(M-L+1\right)}\sum_{t=-T}^{T}\sum_{k=1}^{M-L+1}Z_{k}(n+t)\cdot Z_{k}{}^{H}(n+t) \end{array}$$

where the forward subarray $V_{k}(n)$ and $Z_{k}(n)$ comprise the *k*-th element to the (*k* + *L* − 1)-th element of V(n) and Z(n), respectively. *L* is the length of a subarray in the MV estimation and (2*T* + 1) is the window size for temporal averaging.

In order to further improve the robustness in MV estimation, forward–backward (FB) averaging can be achieved by using the following equation:

(8) $$R_{}^{FB}(n)=\frac{1}{2}(R_{}^{}(n)+JR_{}^{T}(n)J).$$

Note that $JR_{}^{T}(n)J$ represents the backward auto-covariance matrix obtained by converting the forward auto-covariance matrix with the reversal matrix J. Finally, the FB-averaged auto-covariance matrix with diagonal loading is used to calculate the MV weighting vector in (6) as denoted by $w_{RxΜV}^{}(n)$ and $w_{TxΜV}^{}(n)$, respectively, for RxMV and TxMV beamforming. Then, the image output is produced by using the following equation:

(9) $$\begin{array}{l} y_{RxMV}(n)=\frac{1}{N-L+1}\sum_{k=1}^{N-L+1}w_{RxΜV}^{H}(n)V_{k}(n)\\ y_{TxMV}(n)=\frac{1}{M-L+1}\;\sum_{k=1}^{M-L+1}w_{TxΜV}^{H}(n)Z_{k}(n) \end{array}$$

### 2.3. TxMV-DMAS Beamforming for PW Imaging

The image quality of multi-angle PW imaging can be further enhanced by combining the DMAS algorithm with the MV estimation. Using the TxMV beamforming method, for example, the magnitude of the Tx angle array Z(n) is firstly scaled by the *p*-th root while maintaining the same phase to be:

(10) $$\hat{Z}(n)={\left[{\hat{z}}_{1}(n){\hat{z}}_{2}(n){\hat{z}}_{3}(n)\;\ldots {\hat{z}}_{M}(n)\right]}^{T}$$

where

$${\hat{z}}_{i}(n)=\sqrt[{p}]{\left|z_{i}(n)\right|}\cdot e^{j∠\;z_{i}(n)}$$

For the $\hat{Z}(n)$ array, its covariance matrix is estimated with both spatial and temporal averaging and diagonal loading. Then, the weighting vector is calculated using (6) to provide the final image output of TxMV-DMAS beamforming:

(11) $$y_{TxMV-DMAS}(n)={\left[\frac{1}{M-L+1}\;\sum_{k=1}^{M-L+1}{\hat{w}}_{TxΜV}^{H}(n){\hat{Z}}_{k}(n)\right]}^{p}$$

Here, the (∧) represents that both the weighting vector ${\hat{w}}_{TxMV}^{}$ and the *k*-th subarray of the data vector ${\hat{Z}}_{k}(n)$ are obtained with magnitude scaling using the *p*-th root. Therefore, in order to restore the dimensionality of the image output, the *p*-power is performed after the dot product. Moreover, it should be noted that the *p* value determines the degree of spatial coherence included in the DMAS algorithm. Specifically, with *p* = 1, the DMAS algorithm would degenerate into the conventional DAS algorithm.

### 2.4. Adaptive DMAS Beamforming

**Figure 1:**
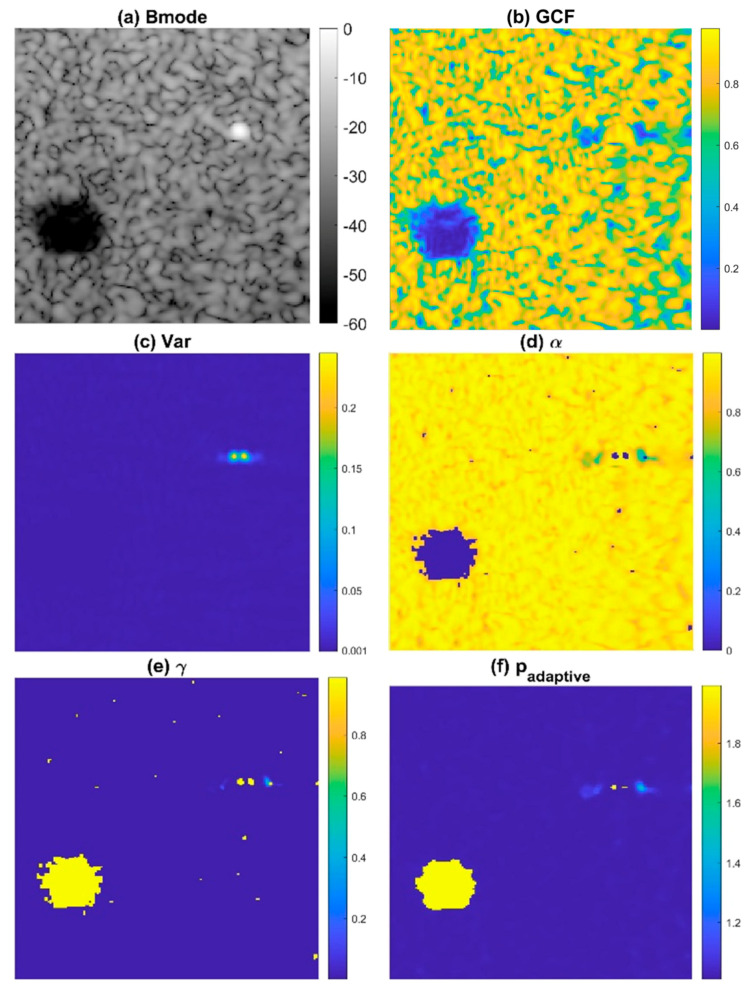
The illustrative (**a**) B-mode images and the corresponding maps of (**b**) GCF, (**c**) Var, (**d**) α(n), and (**e**) γ(n) to determine the final adaptive *p* value (**f**) $p_{adaptive}$. Here, $p_{\max}$ and $p_{\min}$ are 2 and 1, respectively, as an example.

To avoid the degradation of ultrasound speckle quality in conventional DMAS beamforming, we propose the ADMAS algorithm for adaptive selection of the *p* value in the DMAS algorithm. In this study, the adaptive selection of the *p* value in the DMAS algorithm is based on categorizing each image pixel into four distinct types of regions: off-axis clutter near point-like strong reflectors, uncorrelated thermal noises, low-coherence clutter artifacts within the anechoic cyst and speckle background by using the General Coherence Factor (GCF) [26] and the angular variance (Var) in the Tx angle array Z(n) with attenuation compensation. In Figure 1, the B-mode image with an anechoic cyst, wire reflector, and speckle background is demonstrated with the corresponding maps of GCF and Var in the upper panels. It is shown that uncorrelated thermal noises within the cyst have the lowest GCF values, whereas low-coherence clutter artifacts within the cyst have moderate GCF values and low variance. On the contrary, the off-axis clutter region near the point target has a high variance level. These observations indicate that the GCF and Var in combination can be used to identify different regions in ultrasound imaging.

**Figure 2:**
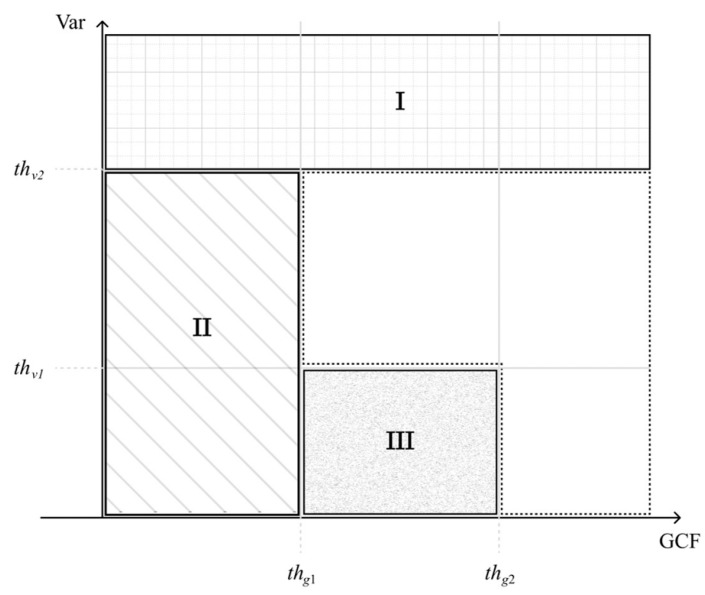
Region categorization of image pixels using GCF and Var. Regions I, II, and III are regarded as having low focusing quality and should correspond to a high *p* value in the adaptive DMAS algorithm.

First, we set the upper bound of thresholding on the signal variance ($th_{v2}$) to identify the off-axis clutter near the wire reflector as region I in Figure 2. In addition, the lower bound of thresholding on the GCF ($th_{g1}$) also helps to identify any uncorrelated noisy pixel that corresponds to region II in Figure 2. Finally, the combination of two threshold values, respectively, on the GCF and the variance ($th_{g2}$ and $th_{v1}$) are used to identify the low-coherence clutter artifacts within the anechoic cyst as region III in Figure 2. Note that the image pixels identified as regions I, II, and III are regarded as having low focusing quality, and thus their GCF value will be reset to 0 for the subsequent selection of the corresponding *p* value.

The aforementioned GCF map is further modified to smooth out its variation in the speckle background region by taking the power according to the signal variance:

(12) $$\alpha (n)=\mathrm{GCF}{\left(n\right)}^{\sqrt[{m}]{\mathrm{Var}(n)}}$$

Note that the signal variance approaches zero in the speckle background as shown in Figure 1c. Therefore, when the signal variance is taken as the power of the original GCF value, the resultant α(n) will be close to the constant value of 1 in the speckle background regardless of the high variation in the original GCF value. Here, the *m*-th root of the signal variance is included to maintain the image contrast of the α(n) map between the speckle background and the anechoic cyst. On the contrary, the α(n) values of image pixels in regions I, II, and III remain zero since their GCF values have been reset to zero. Then, the α(n) value is transformed into the γ(n) value using an inverse S-curve function as follows:

(13) $$\gamma (n)=1-\frac{1}{\sqrt{{1+e}^{-(18\alpha (n)-9)}}}$$

**Figure 3:**
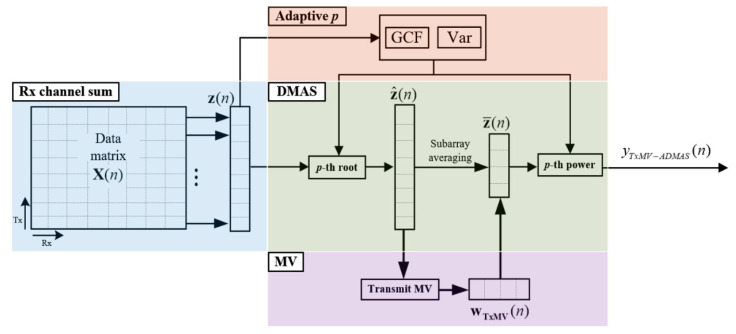
Flow diagram for the proposed TxMV-ADMAS beamforming method.

Note that the purpose of the inverse S-curve is to map low α(n) value to high γ(n) value for subsequent selection of adaptive *p* value. Median filtering is also applied γ(n) in order to eliminate undesired pinholes in the speckle background. Finally, we obtain the adaptive *p* value for each image pixel by using the following equation:

(14) $$p_{adaptive}(n)=\sqrt{\gamma (n)}*(p_{\max}-1)+p_{\min}$$

where $p_{\max}$ and $p_{\min}$, respectively, determine the maximal and the minimal degrees of signal coherence included in the adaptive DMAS beamforming. As shown in Figure 1f, the resultant adaptive *p* value approaches $p_{\max}$ to maximally suppress image pixels inside the anechoic cyst for better image contrast. In contrast, the off-axis clutter near the point-like target also corresponds to $p_{\max}$ in order to provide improvement in image resolution. Note that, however, the adaptive *p* value for the far off-axis clutter region of the point-like target is moderate to avoid the appearance of dark region artifacts. For the speckle background region, on the other hand, the adaptive *p* value will approach $p_{\min}$ to alleviate the deterioration of the speckle quality. The adaptive DMAS method is referred to as the ADMAS method in this study. The flow diagram for the TxMV-ADMAS beamforming method is shown in Figure 3.

## 3. Research Methods

PICMUS datasets established for IEEE IUS 2016 [37] were adopted to evaluate the image quality of multi-angle plane wave imaging. In this study, the performance of the TxMV-DMAS and TxMV-ADMAS beamforming methods are compared using simulated phantoms and experimental phantoms along with in vivo carotid artery in the transverse view. The imaging was conducted using a 128-element linear array that corresponds to *N* = 128 in (1) with a pitch of 0.3 mm, a 2.5-cycle sinusoidal transmit waveform at 5.2 MHz, and a sampling frequency of 20.8 MHz for digitizing the received echoes. PICMUS datasets are acquired using multiple plane wave transmit angles from −16° to +16°, which correspond to *M* = 75 in (1). For receiving, the dynamic aperture size is used with the F-number of 1.75. For spatial smoothing in the direction of the Tx angle of the plane waves, the subarray length is fixed at 25 for all TxMV-based beamforming methods. For RxMV beamforming, however, channel data outside the active receiving aperture were excluded from the estimation of the auto-covariance matrix in the direction of the Rx channel and the subsequent MV estimation. The subarray length for RxMV beamforming was one-third of the size of the active receiving aperture with a minimum value of 2. The diagonal loading parameter was calculated using the equation Δ·trace(R(n)) divided by the subarray size, where Δ is a user-defined factor. Noting this, in DCR-MVDR beamforming, Δ = 1 was used for the simulation of the point-target dataset, and Δ = 5 was used for the speckle dataset as suggested in [19]. For all other MV beamforming methods, Δ = 0.1 was adopted. The temporal averaging in TxMV beamforming was performed with a time window of about 5.4 wavelengths. In our proposed TxMV-ADMAS method, the cutoff frequency $M_{0}$ of GCF estimation [26] was set to 1, and the *m* to adjust the contrast of the α(n) map in (12) was set to 4. Finally, for all PICMUS datasets used in this study, the thresholds for region categorization of each image pixel were empirically determined to be [$th_{g1}$ $th_{g2}$] = [0.1 0.2] for GCF and [$th_{v1}$ $th_{v2}$] = [0.001 0.15] for Var. It is essential to note that the pixel-wise adaptive *p* value was used for the magnitude-scaling of the Z(n) array and also for restoring the signal dimensionality in the proposed TxMV-ADMAS method. The rest of the ADMAS processing remains the same as the conventional DMAS counterpart whose *p* value is a fixed constant for every image pixel.

**Figure 4:**
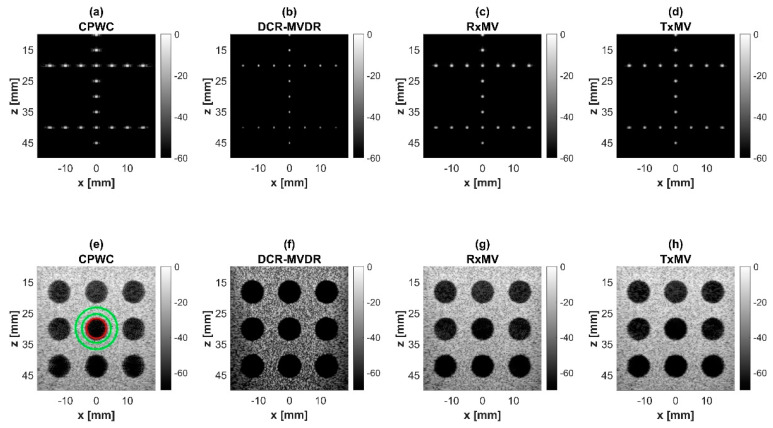
Simulated B-mode images with CPWC, DCR-MVDR, RxMV, and TxMV beamforming methods: (**a**–**d**) are displayed with a dynamic range of 60 dB for the point target dataset, and (**e**–**h**) are displayed with a dynamic range of 70 dB for the anechoic cyst dataset.

In this study, the averaged full width at half maximum (FWHM) of the main lobe of the point targets was used to evaluate the lateral resolution. The contrast radio (CR) and contrast-to-noise-ratio (CNR) were, respectively, calculated using the following two equations:

(15) $$\mathrm{CR}=20\log_{10}(\mu_{bck}/\mu_{cyst})$$

(16) $$\mathrm{CNR}={20\log}_{10}\frac{\left|\mu_{cyst}-\mu_{bck}\right|}{\sqrt{(\sigma_{cyst}{}^{2}+\sigma_{bck}{}^{2})/2}}$$

where $\mu_{cyst}$, $\mu_{bck}$, $\sigma_{cyst}$, and $\sigma_{bck}$ are the mean and the standard deviations of the image magnitude, respectively, of the cyst region (red circle) and the background region (green circle) as indicated in Figure 4 and Figure 13. For both the experimental and simulation datasets, the averaged CR and CNR values for all anechoic cysts are presented in this study. In addition, the generalized contrast-to-noise ratio (gCNR) [38] was also estimated for the experimental results using the probability overlap between the background and cyst regions:

(17) $$\mathrm{gCNR}=1-\int \min\{p_{cyst}(x),p_{bck}(x)\}dx$$

where $p_{cyst}(x)$ and $p_{bck}(x)$ are the probability density functions of the signal envelope in the cyst and the background regions, respectively. Note that the maximal value of gCNR of 1 indicates the complete separation between the background and the cystic lesion.

## 4. Results

In the first part of the results, detailed comparisons among various MV beamforming strategies for multi-angle PW imaging are performed via simulations. It turns out that the TxMV is the optimal MV beamforming in terms of image resolution and contrast. In the second and third parts of the results, the TxMV beamforming is integrated with either conventional DMAS with a global p-value algorithm or the proposed ADMAS with an adaptive *p* value for quantitative evaluation of image quality in simulations and experiments, respectively. The results indicate that compared with the conventional DMAS, the proposed ADMAS helps to restore the image contrast value to the same level of CPWC while the improvement in image resolution remains evident. A detailed demonstration of the complete results is provided as follows.

### 4.1. MV Beamforming without DMAS Algorithm (Simulation)

**Figure 5:**
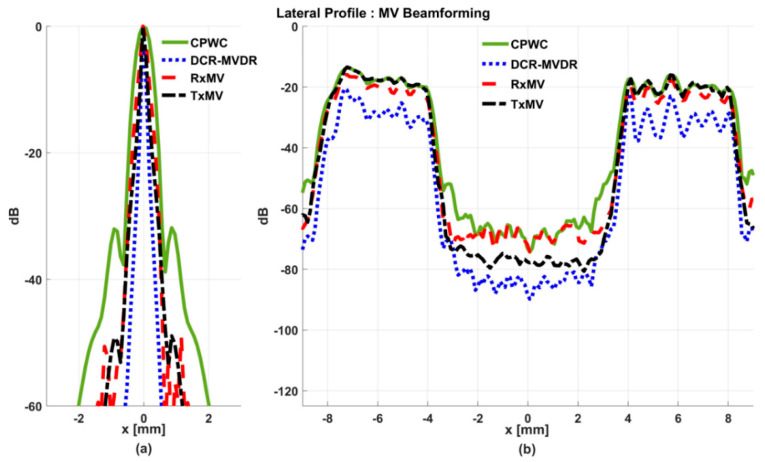
Lateral profiles of the simulated B-mode images in Figure 4 for (**a**) point targets and (**b**) anechoic cysts at the depth of 30 mm.

In this section, simulations are conducted to compare the performance of DCR-MVDR, RxMV, and TxMV beamforming for image resolution and image contrast without including the DMAS algorithm. The CPWC beamforming is considered here as a reference. The simulation dataset for image resolution contains 20-point targets with axial positions ranging from 10 to 45 mm and lateral positions ranging from −15 to 15 mm with a spacing of 5 mm between points. The simulation dataset for image contrast consists of nine anechoic cysts embedded in a speckle background. The corresponding B-mode images are shown in Figure 4 and their lateral profiles at the depth of 30 mm are provided in Figure 5. The corresponding quantitative analysis is in Table 1. The results of simulated B-mode images of point targets and the corresponding lateral profiles indicate that the reference CPWC imaging exhibits the largest main-lobe FWHM of 0.57 mm and significant off-axis clutter in the lateral direction. While both DCR-MVDR and TxMV beamforming provide a comparable improvement in the main-lobe FWHM of point targets to 0.16 and 0.17 mm, respectively, DCR-MVDR beamforming appears to suffer from loss of signal intensity on the left and right boundaries of the B-mode image. On the contrary, RxMV beamforming can only provide moderate improvement in the main-lobe FWHM to 0.28 mm.

On the other hand, the B-mode results of simulated images of anechoic cysts and the corresponding lateral profiles also indicate that MV beamforming is capable of suppressing the marked image clutters within the anechoic cysts in CPWC imaging. For example, DCR-MVDR imaging effectively suppresses the level of clutter artifacts within the anechoic cyst and hence improves the CR from 40.1 dB of CPWC to 47.4 dB. However, due to the lack of spatial averaging, the speckle background suffers from the granular appearance and also the decrease in background intensity. Consequently, even though the CR value of DCR-MVDR imaging is noticeably higher than the CPWC, the CNR decreases considerably from 16.0 of CPWC to 14.5. Note that the lower CNR may affect the detection of low-contrast lesions in clinical scenarios. RxMV beamforming is capable of maintaining the speckle quality but its clutter suppression is inferior to DCR-MVDR. In contrast, the speckle quality of TxMV beamforming is also comparable to that of CPWC while the clutter suppression is as effective as DCR-MVDR. This is consistent with the lateral profiles of the anechoic cyst in Figure 5, which shows that the difference in image magnitude between the cyst and the background is comparable to DCR-MVDR and TxMV beamforming. The lateral profiles of the anechoic cyst also demonstrate that TxMV beamforming better maintains the signal level in the speckle background and avoids the speckle variation compared with the DCR-MVDR counterpart. Consequently, TxMV beamforming leads to the highest CR of 47.8 and the highest CNR of 17.7, indicating that TxMV beamforming outperforms other methods in the tradeoff of image resolution and image contrast. Therefore, in this study, the TxMV beamforming method is adapted to combine it with the DMAS algorithm to further enhance the image quality. This is also why only the TxMV-DMAS beamforming method is introduced in detail in Section 2.

### 4.2. MV Beamforming with DMAS Algorithm (Simulation)

**Figure 6:**
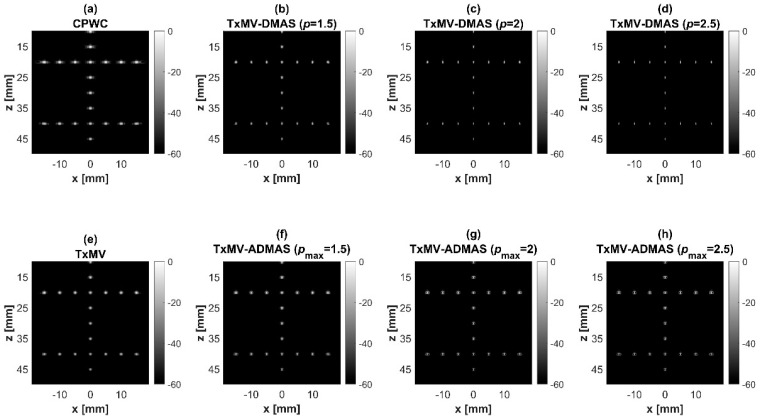
Simulated B-mode images for the point target dataset using (**a**) CPWC, (**e**) TxMV, (**b**–**d**) TxMV-DMAS, and (**f**–**h**) TxMV-ADMAS beamforming methods. The *p* values in the DMAS algorithm and the maximal *p* values in the ADMAS algorithm are 1.5, 2, and 2.5, respectively, from (**b**–**d**) and (**f**–**h**).

**Figure 7:**
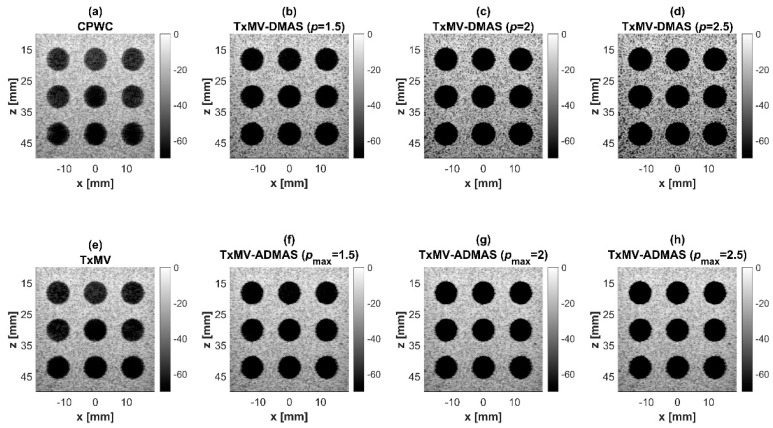
Simulated B-mode images for the anechoic cyst dataset with (**a**) CPWC, (**e**) TxMV, (**b**–**d**) TxMV-DMAS, and (**f**–**h**) TxMV-ADMAS beamforming methods. The *p* values in the DMAS algorithm and the maximal *p* values in the ADMAS algorithm are 1.5, 2, and 2.5, respectively, from (**b**–**d**) and (**f**–**h**).

**Figure 8:**
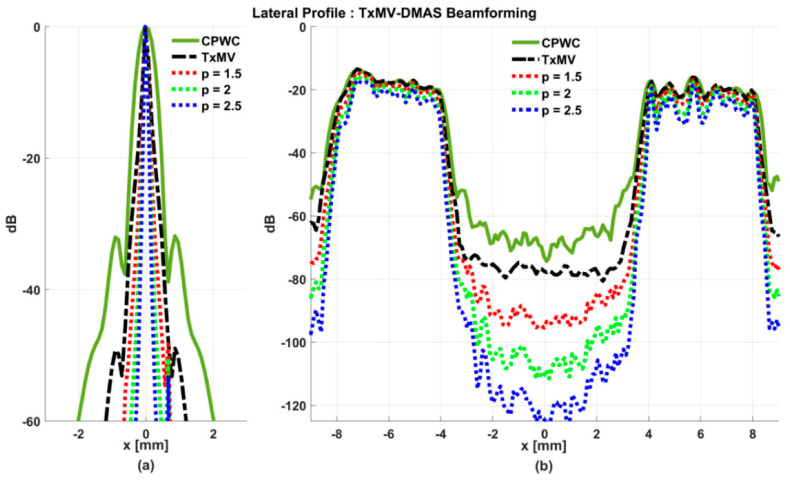
Lateral profiles of simulated B-mode images with TxMV-DMAS beamforming for (**a**) point targets in Figure 6 and (**b**) anechoic cysts in Figure 7 at the depth of 30 mm.

Simulated B-mode images of TxMV-DMAS beamforming as a function of the *p* value are shown in the upper panels of Figure 6 and Figure 7, respectively, for the point target and anechoic cyst datasets. CPWC and TxMV beamforming are also provided for comparison. Note that TxMV beamforming is equivalent to TxMV-DMAS when *p* = 1. The lateral profiles at the depth of 30 mm are provided in Figure 8. The corresponding quantitative analysis of image resolution and image contrast are also demonstrated in Table 2. It can be observed from the B-mode images of point targets in Figure 6b–d that the main-lobe width of TxMV-DMAS is significantly reduced with increasing *p* value. Lateral profiles in Figure 8a also demonstrate that the increase in *p* value in the DMAS algorithm not only reduces the main-lobe width but also effectively suppresses off-axis clutters. For example, as shown in Table 2, the main-lobe FWHM in TxMV-DMAS beamforming decreases from 0.17 mm to 0.07 mm when the *p* value increases from 1 to 2.5. These results confirm that the DMAS algorithm can effectively improve image resolution in TxMV beamforming. On the other hand, B-mode images of anechoic cysts in Figure 7b–d also show that the clutter artifacts within the cysts are significantly reduced when the *p* value increases.

Using the lateral profiles in Figure 8b, it can be readily observed that the level of clutter artifacts within the cyst decreases with the *p* value together with a sharper edge of the anechoic cyst due to the improved lateral resolution. However, the downside of increasing the *p* value in the DMAS algorithm is the reduction in the signal intensity and the smoothness of the speckle background. Consequently, the speckle background of the B-mode image appears darker and more granular with a higher *p* value in TxMV-DMAS beamforming. According to the corresponding quantitative analysis in Table 2, TxMV-DMAS beamforming is capable of improving the CR value from 47.8 dB to 75.8 dB when the *p* value increases from 1 to 2.5 while the corresponding CNR adversely decreases from 17.7 to 15.4. Note that the CNR of TxMV-DMAS beamforming with *p* = 2.5 was even lower than that of conventional CPWC beamforming, indicating the compromised contrast detection in the presence of a granular speckle background.

**Figure 9:**
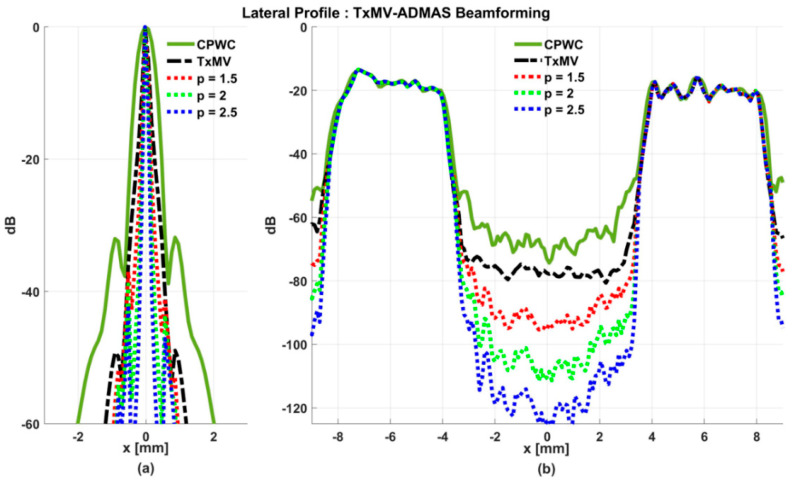
Lateral profiles of simulated B-mode images with TxMV-ADMAS beamforming for (**a**) point targets in Figure 6 and (**b**) anechoic cysts in Figure 7 at the depth of 30 mm.

The limitation of TxMV-DMAS beamforming actually comes from the fact that a fixed *p* value in the DMAS algorithm is adopted regardless of the different characteristics of the imaged region. This limitation would be alleviated by adopting the ADMAS algorithm to provide an adaptive *p* value for each image pixel. Simulated B-mode images of TxMV-ADMAS beamforming as a function of *p* value are demonstrated in Figure 6f–h for point targets. As shown in Table 2, TxMV-ADMAS beamforming provides a comparable image resolution to TxMV-DMAS in terms of main-lobe FWHM, though their point spread functions look different. This is simply because, in TxMV-ADMAS beamforming, a smaller *p* value is assigned to partially coherent pixels in the off-axis clutter region to avoid excessive suppression. Figure 9a also confirms that the lateral profiles of point targets in TxMV-ADMAS beamforming markedly differ from those in TxMV-DMAS beamforming for partially coherent regions such as near side-lobe clutter within ±1 mm in the lateral position. Therefore, it is expected that the main-lobe FWHM in TxMV-ADMAS remains similar to that in TxMV-DMAS. On the other hand, simulated B-mode images of TxMV-ADMAS beamforming for anechoic cysts in Figure 7f–h and the corresponding lateral profiles in Figure 9b demonstrate that TxMV-ADMAS not only suppresses the image clutters to the same level as the TxMV-DMAS counterpart but also maintains the speckle quality of the background. For example, the lateral profiles indicate that the speckle background on both sides of the cyst remains unchanged with the *p* value so that both the speckle magnitude and the variation are comparable to the CPWC image. As a result, TxMV-ADMAS beamforming outperforms the TxMV-DMAS counterpart by providing a higher CNR with comparable CR.

### 4.3. MV Beamforming with DMAS Algorithm (Experiments)

**Figure 10:**
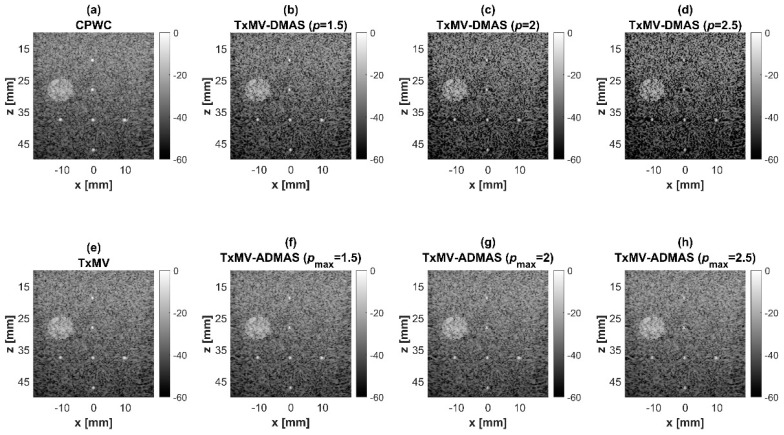
Experimental B-mode images for the point target dataset with (**a**) CPWC, (**e**) TxMV, (**b**–**d**) TxMV-DMAS, and (**f**–**h**) TxMV-ADMAS beamforming methods. The *p* values in the DMAS algorithm and the maximal *p* values in the ADMAS algorithm are 1.5, 2, and 2.5, respectively, from (**b**–**d**) and (**f**–**h**).

**Figure 11:**
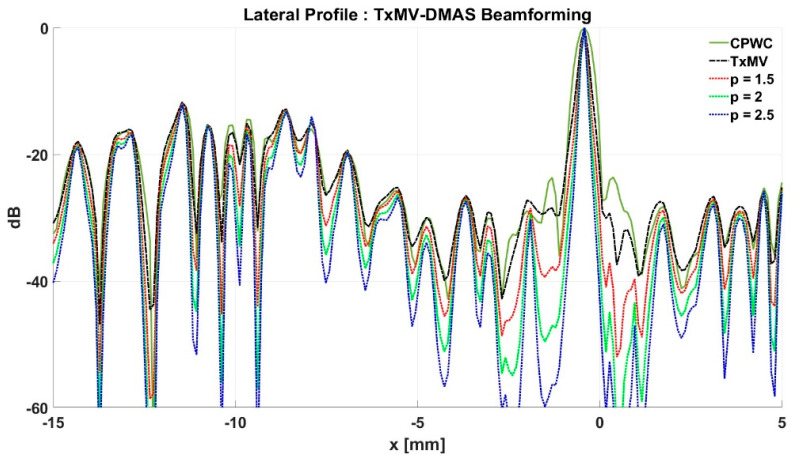
Lateral profiles of simulated B-mode images with TxMV-DMAS beamforming in Figure 10 at the depth of 28 mm.

**Figure 12:**
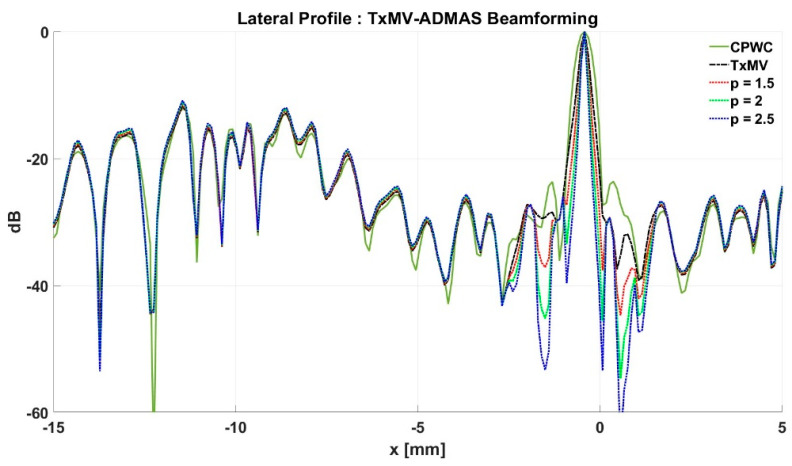
Lateral profiles of simulated B-mode images with TxMV-ADMAS beamforming in Figure 10 at the depth of 28 mm.

To further investigate the performance of our proposed beamforming methods, PICMUS experimental datasets obtained from scanning a multi-purpose tissue phantom were also considered. The B-mode images of wire targets for analyzing image resolution are shown in Figure 10 and the quantitative main-lobe FWHM are presented in Table 3. Similar to the simulation results, TxMV without the DMAS algorithm improves the image resolution by reducing the FWHM from 0.57 mm of CPWC to 0.36 mm. When the DMAS algorithm is integrated with the TxMV beamforming, the main lobe of wire targets in the resultant TxMV-DMAS becomes narrower with increasing *p* value. For example, the main-lobe FWHM reduces to 0.28, 0.24, and 0.21 mm when *p* is 1.5, 2.0, and 2.5, respectively, in TxMV-DMAS beamforming. However, the B-mode images of TxMV-DMAS showed evident black-wing artifacts on both sides of the wire because a fixed *p* value is adopted for every image pixel. The hyperechoic cyst also suffers from elevated speckle variation, which compromises its detectability from the background region especially when the *p* value is large. This is clearly demonstrated by the lateral profiles of TxMV-DMAS at the depth of 28 mm in Figure 11. Conversely, the adaptive selection of the *p* value in TxMV-ADMAS helps to alleviate the black-wing artifact of wires while the image resolution remains comparable to that of TxMV-DMAS with the same *p* value. It should be noted that the TxMV-ADMAS also reduces the speckle variation in the B-mode image of the tissue phantom so that the hyperechoic cyst in Figure 10 can be better detected from the background. Figure 12 shows the corresponding lateral profiles of TxMV-ADMAS. The results indicate that the black-wing artifacts on the wires were significantly alleviated by the adaptive selection of the *p* value together with improved image resolution and comparable speckle pattern of the CPWC reference.

**Figure 13:**
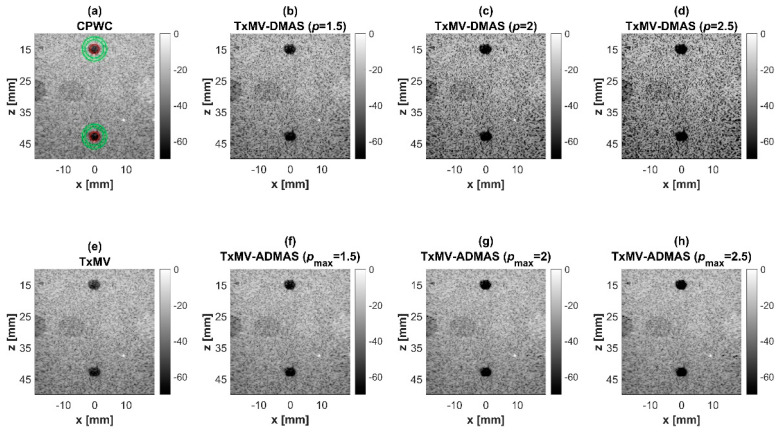
Experimental B-mode images for the anechoic cyst dataset with (**a**) CPWC, (**e**) TxMV, (**b**–**d**) TxMV-DMAS, and (**f**–**h**) TxMV-ADMAS beamforming methods. The *p* values in the DMAS algorithm and the maximal *p* values in the ADMAS algorithm are 1.5, 2, and 2.5, respectively from (**b**–**d**) and (**f**–**h**).

On the other hand, the experimental dataset for image contrast consists of anechoic cysts located at depths of about 15 and 45 mm as shown in Figure 13. Quantitative image contrast metrics for cystic lesions in Table 3 include not only the CR and the CNR but also the additional gCNR. The experimental results showed that TxMV has difficulties coping with complex experimental environments and thus barely improves the image contrast as compared with CPWC. This is evident because significant clutter artifacts inside the cysts remain unsuppressed in TxMV beamforming. On the contrary, TxMV-DMAS markedly improves the image CR due to the effective removal of the clutter artifacts inside the cyst with increasing *p* value. Nonetheless, similar to the observations from simulations, the speckle background of TxMV-DMAS not only decreases in magnitude but also suffers from granular appearance. Consequently, when *p* increases from 1 to 2.5 in TxMV-DMAS beamforming, the CNR and the gCNR in Table 3 decrease from 13.0 to 10.9 and from 0.97 to 0.91, respectively. These results indicate that, although TxMV-DMAS is capable of higher image contrast, the detectability of cystic lesions in the speckle background may be compromised due to the loss of speckle quality. In contrast, TxMV-ADMAS with adaptive *p* value could provide similar image contrast to TxMV-DMAS while keeping the speckle background undamaged. Therefore, TxMV-ADMAS consistently leads to a higher CNR than the TxMV-DMAS counterpart with the same *p* value.

**Figure 14:**
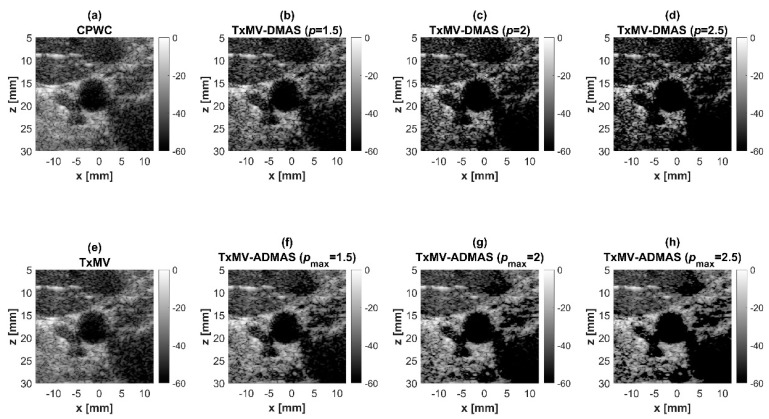
Cross-sectional view of in vivo carotid images generated with different beamformers: (**a**) CPWC, (**e**) TxMV, (**b**–**d**) TxMV-DMAS, and (**f**–**h**) TxMV-ADMAS. The maximal *p* values in DMAS beamforming are 1.5, 2, and 2.5, respectively.

An in vivo dataset of PICMUS was also examined in this study to evaluate the robustness of the MV estimation in clinical scenarios. The B-mode images of the carotid artery of a healthy volunteer are shown in Figure 14. Generally, similar to the experimental results on the tissue-mimicking phantom, the CPWC and TxMV suffer from obvious clutter within the carotid artery for the in vivo dataset. On the contrary, both TxMV-DMAS and TxMV-ADMAS demonstrate effective clutter suppression within the carotid artery. Nonetheless, speckle quality in the tissue background is clearly damaged with reduced image magnitude when the fixed *p* value in TxMV-DMAS increases. In the adaptive DMAS algorithm, however, a higher *p* value will be adopted to suppress the acoustic clutter within the carotid artery while the tissue background is better preserved by a lower *p* value. Meanwhile, the image resolution remains effectively improved in TxMV-ADMAS due to the MV estimation in beamforming.

## 5. Discussions and Conclusions

In this study, we proposed the combination of the MV beamforming method with the DMAS algorithm to provide effective improvement in both resolution and contrast of multi-angle PW imaging. Compared with our preliminary results in [35], the contributions in this study are (1) detailed examinations of various MV beamforming strategies for multi-angle PW imaging, (2) adaptive selection of pixel-wise *p* values in the DMAS algorithm, and (3) the integration of optimal MV beamforming with the adaptive DMAS algorithm to improve image resolution and contrast without sacrificing speckle quality. The results indicate that the TxMV method outperforms the other MV beamforming candidates in terms of image resolution and image contrast. Subsequently, TxMV beamforming was integrated with the proposed ADMAS algorithm for further enhancement of image quality. The resultant TxMV-ADMAS beamforming method was demonstrated to restore the image contrast value to the same level as CPWC while the improvement in image resolution remained evident. To determine the optimal MV beamforming method, it should be noted that the multi-angle PW imaging is actually constructed using the 2D echo matrix with the PW transmit angle and the receiving channel in each dimension. Therefore, various MV beamforming methods have been proposed specifically for multi-angle PW imaging to exploit the 2D echo matrix in different ways for the estimation of MV weight and the subsequent MV image output. Among them, DCR-MVDR beamforming stands out by generating the snapshots for the covariance matrix estimation from a set of Tx vectors. Each Tx vector is obtained by compounding the 2D echo matrix in the receiving channel but excluding one particular channel in the sum. Since DCR-MVDR utilizes the full size of the 2D echo matrix for decorrelation, it is reported to outperform conventional methods such as double MV and JTR MV, which employ spatial averaging of the covariance matrix. However, the implementation of DCR-MVDR beamforming could be limited by its huge memory requirement for the storage of the entire 2D echo matrix per image pixel and the computationally demanding inversion of the full-size covariance matrix. On the contrary, for better memory efficiency, the 2D echo matrix can be first compounded along either the Tx or Rx direction to acquire the corresponding one-dimensional Rx channel array or Tx angle array. Then, subsequent MV estimation can be applied to the one-dimensional Rx channel array or the Tx angle array as the RxMV or TxMV beamforming methods, respectively, in this study.

In the first part of our study, in order to choose the optimal MV beamforming method for multi-angle PW imaging, the RxMV and TxMV methods were compared with the DCR-MVDR for their performance without the DMAS algorithm. The simulation results indicated that the DCR-MVDR exhibits significant improvements in image resolution and contrast but suffers from a considerable degradation in CNR. This effect is likely due to the absence of subarray averaging. Specifically, though a larger length of subarray leads to notable enhancement in image resolution, the corresponding smaller number of subarray averaging would compromise both the intensity and smoothness of the speckle background [39]. Since the DCR-MVDR can be understood to estimate the covariance matrix using the maximum length of the subarray, it is reasonable to expect the image resolution of DCR-MVDR to be gained by sacrificing the speckle quality. On the contrary, TxMV beamforming performs comparably to the DCR-MVDR counterpart in terms of image resolution with markedly higher speckle quality while the performance of RxMV is consistently inferior to that of TxMV in all aspects. Since a fixed F-number of 1.75 is used in this study for reception, the number of active elements available varies with the depth of each image pixel and thus temporal averaging is not applicable to the RxMV method. This may explain the loss of speckle quality in RxMV compared with TxMV. Moreover, the limitation on active elements would also decrease the length of the subarray for RxMV, especially for image pixels at shallower depth, which reduces the resolution and contrast of the entire RxMV image. Therefore, it appears that the TxMV beamforming method outperforms the other two candidates of MV beamforming in the tradeoff of image resolution and image contrast and thus was combined with the DMAS algorithm to further enhance its image quality. Nonetheless, it should be noted that the improvement in image resolution due to MV estimation alone (i.e., TxMV) is relatively less in Table 3 for the experimental dataset than in Table 2 for the simulation dataset. This occurs when the main-lobe signal deviates from its original direction due to the presence of acoustic reverberations in practical scenarios such that the optimization of MV weighting could be compromised.

Compared with the other MV-DMAS beamforming method proposed for multi-angle PW imaging [40], in which the DMAS algorithm is sequentially applied to the Tx angle data after MV estimation in the receiving channel, the proposed TxMV-DMAS beamforming method is performed by integrating the DMAS algorithm into the MV estimation such that the MV estimation would benefit from the improved signal coherence from the DMAS algorithm. The resultant TxMV-DMAS beamforming demonstrates superior image resolution and image contrast compared with TxMV without DMAS in both simulations and experiments. However, the signal coherence-based DMAS algorithm inevitably elevates the speckle variation and thus compromises the corresponding speckle quality and the achievable CNR. Note that the decrease in speckle quality has also been reported in Tx-DMAS imaging without MV estimation [36]. These observations indicate that since the diffused scattering in the speckle background intrinsically exhibits random variation in signal coherence and the corresponding image magnitude, the DMAS algorithm tends to exaggerate the speckle variation by further applying coherence-based weighting to the image output. This explains why the speckle region in DMAS beamforming would suffer from more variation in magnitude than conventional CPWC beamforming.

In response to the drawbacks of the conventional DMAS algorithm, it is essential to adaptively determine the *p* value in the DMAS algorithm according to the characteristics of the image pixel. For the pixel in the speckle background region, the *p* value is required to approach 1 to maintain the speckle quality. For the pixel suffering from severe clutter artifacts or thermal noises, on the contrary, the *p* value should be high to provide improved image resolution and contrast. In this study, different image regions are categorized using the GCF and the signal variance in the Tx angle array for the adaptive selection of the *p* value in the DMAS algorithm. The experimental results demonstrate that the proposed ADMAS algorithm in TxMV beamforming (i.e., TxMV-ADMAS beamforming) is capable of providing higher *p* values in the clutter region to achieve similar image resolution and contrast as TxMV-DMAS. Meanwhile, *p* values close to 1 are used in the speckle region and result in a speckle quality close to that of DAS beamforming. Consequently, the TxMV-ADMAS beamforming method is able to provide higher CNR and gCNR compared with TxMV-DMAS. It should be also noted that, in the proposed ADMAS algorithm, the set of threshold values for the GCF and the signal variance for categorizing the image regions remains unchanged for both the simulation and the experimental datasets. In other words, the criterion to identify different image regions using the GCF and the signal variance in multi-angle PW imaging could be universal and thus applicable to a wide range of imaging scenarios. Nonetheless, as shown in Figure 13, the proposed TxMV-ADMAS is particularly effective in suppressing clutter artifacts in anechoic cysts and thus provides more visual contrast in the detection of anechoic cysts than the echogenic cysts with the increasing *p* value. Based on this observation, the proposed method would be relatively beneficial in detecting vessels and heart chambers in ultrasound imaging.

Nonetheless, when the PW transmit angles involved in the Tx angles array are different, it is expected that the threshold values in this study should be re-optimized to consider the change in transmit condition. Specifically, since the GCF represents the angular coherence of the image pixel among different Tx angles, its baseline value for the speckle background would inevitably change with the PW transmit angles at which the angular response of the image pixel is sampled. This is particularly true when the number of PW transmit angles is limited to achieve a high frame rate in the applications of ultrafast transient elastography. Another possible limitation of the proposed TxMV-ADMAS beamforming method would be its high computational complexity. Recall that the MV estimation comprises two major steps for each image pixel: subarray and temporal averaging of the covariance matrix and the estimation of MV weighting. According to (6) and (7), the averaging of the covariance matrix in TxMV beamforming involves $O((2T+1)\cdot (M-L+1)\cdot {\left(L\right)}^{2})$ complex operations and the estimation of MV weighting takes $O((L{)}^{3})$ complex operations for the inverse of the covariance matrix. When the computational complexity is not acceptable due to the limitation on hardware in low-end imaging systems, the suboptimal solution would be to remove the MV estimation from the proposed TxMV-ADMAS beamforming to provide only Tx-ADMAS beamforming. Note that Tx-ADMAS beamforming will also benefit from the pixel-wise adaptive *p* value due to the improvement in the background speckle quality and the suppression of clutter artifacts even without MV estimation. In the case of Tx-ADMAS beamforming, moreover, its computational complexity can be significantly reduced to a comparable level similar to the conventional CPWC beamforming (i.e., O(M)). An alternative solution to reduce the computation burden of the proposed TxMV-ADMAS beamforming method is the adoption of kernel-based MV weighting as suggested in [41]. Specifically, the covariance matrix is constructed from all pixels in the kernel such that the resultant MV weighting is derived from only one matrix inversion and is then applied to all pixels in the corresponding kernel. This will be examined in our future work on the TxMV-ADMAS beamforming method.

## Figures and Tables

**Table 1:** Image quality metrics of MV beamformer without DMAS algorithm using PICMUS simulation datasets.

Beamformer	FWHM (mm)	CR (dB)	CNR
CPWC	0.57	40.1	16.0
DCR-MVDR	0.16	47.4	14.5
RxMV	0.28	43.4	16.6
TxMV	0.17	47.8	17.7

**Table 2:** Image quality metrics of TxMV-DMAS and TxMV-ADMAS beamforming using PICMUS simulation datasets.

*p* Value	Beamformer	FWHM (mm)	CR (dB)	CNR
1	CPWC	0.57	40.1	16.0
	TxMV	0.17	47.8	17.7
1.5	TxMV-DMAS	0.12	58.3	16.8
	TxMV-ADMAS	0.11	59.6	17.9
2	TxMV-DMAS	0.09	67.2	16.0
	TxMV-ADMAS	0.09	69.0	17.7
2.5	TxMV-DMAS	0.07	75.8	15.4
	TxMV-ADMAS	0.09	77.6	17.5

**Table 3:** Image quality metrics of TxMV-DMAS and TxMV-ADMAS beamforming using PICMUS experimental datasets.

*p* Value	Beamformer	FWHM (mm)	CR (dB)	CNR	gCNR
1	CPWC	0.57	27.0	12.8	0.97
	TxMV	0.36	27.4	13.0	0.97
1.5	TxMV-DMAS	0.28	33.2	11.9	0.94
	TxMV-ADMAS	0.28	33.4	13.0	0.97
2	TxMV-DMAS	0.24	38.0	11.3	0.92
	TxMV-ADMAS	0.23	37.3	12.8	0.97
2.5	TxMV-DMAS	0.21	42.1	10.9	0.91
	TxMV-ADMAS	0.20	39.6	12.5	0.97

## Data Availability

PICMUS datasets are open-access datasets.

## References

[1] Sandrin L., Catheline S., Tanter M., Hennequin X., Fink M. (1999). Time-resolved pulsed elastography with ultrafast ultrasonic imaging. Ultrason. Imaging.

[2] So H., Chen J., Yiu B., Yu A. (2011). Medical ultrasound imaging: To GPU or not to GPU?. IEEE Micro.

[3] Tanter M., Fink M. (2014). Ultrafast imaging in biomedical ultrasound. IEEE Trans. Ultrason. Ferroelectr. Freq. Contr..

[4] Montaldo G., Tanter M., Bercoff J., Benech N., Fink M. (2009). Coherent plane-wave compounding for very high frame rate ultrasonography and transient elastography. IEEE Trans. Ultrason. Ferroelectr. Freq. Contr..

[5] Foiret J., Cai X., Bendjador H., Park E.Y., Kamaya A., Ferrara K.W. (2022). Improving plane wave ultrasound imaging through real-time beamformation across multiple arrays. Sci. Rep..

[6] Zhou Z., Wang Y., Yu J., Guo Y., Guo W., Qi Y. (2018). High spatial–temporal resolution reconstruction of plane-wave ultrasound images with a multichannel multiscale convolutional neural network. IEEE Trans. Ultrason. Ferroelectr. Freq. Contr..

[7] Luchies A.C., Byram B.C. (2018). Deep neural networks for ultrasound beamforming. IEEE Trans. Med. Imag..

[8] Tang J., Zou B., Li C., Feng S., Peng H. (2021). Plane-wave image reconstruction via generative adversarial network and attention mechanism. IEEE Trans. Instrum. Meas..

[9] Lu J., Millioz F., Garcia D., Salles S., Ye D., Friboulet D. (2022). Complex convolutional neural networks for ultrafast ultrasound imaging reconstruction from in-phase/quadrature signal. IEEE Trans. Ultrason. Ferroelectr. Freq. Contr..

[10] Lu J.Y., Lee P.Y., Huang C.C. (2022). Improving image quality for single-angle plane wave ultrasound imaging with convolutional neural network beamformer. IEEE Trans. Ultrason. Ferroelectr. Freq. Contr..

[11] Synnevag J.F., Austeng A., Holm S. (2007). Adaptive beamforming applied to medical ultrasound imaging. IEEE Trans. Ultrason. Ferroelectr. Freq. Contr..

[12] Synnevag J.F., Austeng A., Holm S. (2009). Benefits of minimum-variance beamforming in medical ultrasound imaging. IEEE Trans. Ultrason. Ferroelectr. Freq. Contr..

[13] Nilsen C.I.C., Hafizovic I. (2009). Beamspace adaptive beamforming for ultrasound imaging. IEEE Trans. Ultrason. Ferroelectr. Freq. Contr..

[14] Asl B.M., Mahloojifar A. (2010). Eigenspace-based minimum variance beamforming applied to medical ultrasound imaging. IEEE Trans. Ultrason. Ferroelectr. Freq. Contr..

[15] Zeng X., Chen C., Wang Y. (2012). Eigenspace-based minimum variance beamformer combined with Wiener postfilter for medical ultrasound imaging. Ultrasonics..

[16] Mehdizadeh S., Austeng A., Johansen T.F., Holm S. (2012). Eigenspace based minimum variance beamforming applied to ultrasound imaging of acoustically hard tissues. IEEE Trans. Med. Imag..

[17] Zhao J., Wang Y., Zeng X., Yu J., Yiu B.Y.S., Yu A.C.H. (2015). Plane wave compounding based on a joint transmitting-receiving adaptive beamformer. IEEE Trans. Ultrason. Ferroelectr. Freq. Contr..

[18] Rindal O.M.H., Austeng A. Double adaptive plane-wave imaging. Proceedings of the 2016 IEEE International Ultrasonics Symposium.

[19] Nguyen N.Q., Prager R.W. (2018). A spatial coherence approach to minimum variance beamforming for plane-wave compounding. IEEE Trans. Ultrason. Ferroelectr. Freq. Contr..

[20] Wang S.L., Li P.C. (2009). MVDR-based coherence weighting for high-frame-rate adaptive imaging. IEEE Trans. Ultrason. Ferroelectr. Freq. Contr..

[21] Asl B.M., Mahloojifar A. (2009). Minimum variance beamforming combined with adaptive coherence weighting applied to medical ultrasound imaging. IEEE Trans. Ultrason. Ferroelectr. Freq. Contr..

[22] Qi Y., Wang Y., Guo W. (2018). Joint subarray coherence and minimum variance beamformer for multitransmission ultrasound imaging modalities. IEEE Trans. Ultrason. Ferroelectr. Freq. Contr..

[23] Nilsen C.I., Holm S. (2010). Wiener beamforming and the coherence factor in ultrasound imaging. IEEE Trans. Ultrason. Ferroelectr. Freq. Contr..

[24] Paridar R., Asl B.M. (2023). Plane wave ultrasound imaging using compressive sensing and minimum variance beamforming. Ultrasonics..

[25] Hollman K.W., Rigby K.W., Donnell M.O. Coherence factor of speckle from a multi-row probe. Proceedings of the 1999 IEEE Ultrasonics Symposium.

[26] Li P.C., Li M.L. (2003). Adaptive imaging using the generalized coherence factor. IEEE Trans. Ultrason. Ferroelectr. Freq. Contr..

[27] Lediju M.A., Trahey G.E., Byram B.C., Dahl J.J. (2011). Short-lag spatial coherence of backscattered echoes: Imaging characteristics. IEEE Trans. Ultrason. Ferroelectr. Freq. Contr..

[28] Pinton G., Trahey G.E., Dahl J.J. (2014). Spatial coherence in human tissue: Implications for imaging and measurement. IEEE Trans. Ultrason. Ferroelectr. Freq. Contr..

[29] Matrone G., Savoia A.S., Caliano G., Magenes G. (2015). The delay multiply and sum beamforming algorithm in ultrasound B-mode medical imaging. IEEE Trans. Med. Imag..

[30] Matrone G., Ramalli A. (2018). Spatial coherence of backscattered signals in multi-line transmit ultrasound imaging and its effect on short-lag filtered-delay multiply and sum beamforming. Appl. Sci..

[31] Shen C.C., Hsieh P.Y. (2019). Ultrasound baseband delay-multiply-and-sum (BB-DMAS) nonlinear beamforming. Ultrasonics.

[32] Polichetti M., Varray F., Béra J.C., Cachard C., Nicolas B. (2018). A nonlinear beamformer based on p-th root compression—Application to plane wave ultrasound imaging. Appl. Sci..

[33] Prieur F., Rindal O.M.H., Austeng A. (2018). Signal coherence and image amplitude with the filtered delay multiply and sum beamformer. IEEE Trans. Ultrason. Ferroelectr. Freq. Contr..

[34] Shen C.C. (2021). Computationally efficient minimum-variance baseband delay-multiply-and-sum beamforming for adjustable enhancement of ultrasound image resolution. Ultrasonics.

[35] Huang G.L., Shen C.C. Delay-multiply-and-sum beamforming with transmit minimum-variance estimation in multi-angle plane-wave imaging. Proceedings of the 2022 IEEE International Ultrasonics Symposium.

[36] Shen C.C., Hsieh P.Y. (2019). Two-dimensional spatial coherence for ultrasonic DMAS beamforming in multi-angle plane-wave imaging. Appl. Sci..

[37] Liebgott H., Rodriguez-Molares A., Cervenansky F., Jensen J.A., Bernard O. Plane-wave imaging challenge in medical ultrasound. Proceedings of the 2016 IEEE International Ultrasonics Symposium.

[38] Rodriguez-Molares A., Rindal O.M.H., D’Hooge J., Måsøy S.E., Austeng A., Lediju Bell M.A., Torp H. (2020). The generalized contrast-to-noise ratio: A formal definition for lesion detectability. IEEE Trans. Ultrason. Ferroelectr. Freq. Contr..

[39] Synnevåg J.F., Nilsen C.I.C., Holm S. Speckle statistics in adaptive beamforming. Proceedings of the 2007 IEEE International Ultrasonics Symposium.

[40] Ziksari M.S., Asl B.M. (2021). Minimum variance combined with modified delay multiply-and-sum beamforming for plane-wave compounding. IEEE Trans. Ultrason. Ferroelectr. Freq. Contr..

[41] Hashemseresht M., Afrakhteh S., Behnam H. (2022). High-resolution and high-contrast ultrafast ultrasound imaging using coherent plane wave adaptive compounding. Biomed. Signal Process. Control.

